# Rheological properties of corn stover slurries during fermentation by *Clostridium thermocellum*

**DOI:** 10.1186/s13068-018-1248-z

**Published:** 2018-09-08

**Authors:** Sanchari Ghosh, Evert K. Holwerda, Robert S. Worthen, Lee R. Lynd, Brenden P. Epps

**Affiliations:** Thayer School of Engineering, Dartmouth College, Hanover, NH USA

**Keywords:** Biomass, Corn stover, Rheology, Slurry viscosity, Large amplitude oscillatory shear, Cotreatment, Consolidated bioprocessing, *Clostridium thermocellum*, Bioenergy

## Abstract

**Background:**

Milling during fermentation, termed cotreatment, has recently been proposed as an alternative to thermochemical pretreatment as a means to increase the accessibility of lignocellulosic biomass to biological attack. A central premise of this approach is that partial solubilization of biomass changes the slurry’s physical properties such that milling becomes more impactful and more feasible. A key uncertainty is the energy required to mill partially fermented biomass. To inform both of these issues, we report rheological characterization of small-particle, corn stover slurries undergoing fermentation by *Clostridium thermocellum*.

**Results:**

Fermented and unfermented corn stover slurries were found to be shear-thinning and well described by a power law model with an exponent of 0.10. Plastic viscosity of a slurry, initially at 16 wt.% insoluble solids, decreased as a result of fermentation by a factor of 2000, with the first eightfold reduction occurring in the first 10% of carbohydrate conversion. Large amplitude oscillatory shear experiments revealed only minor changes to the slurry’s rheological fingerprint as a result of fermentation, with the notable change being a reduction in the critical strain amplitude needed for the onset of nonlinearity. All slurries were found to be elastoviscoplastic, with the elastic/viscous crossover at roughly 100% strain amplitude.

**Conclusions:**

Whereas prior biomass rheology studies have involved pretreated feedstocks and solubilization mediated by fungal cellulase, we report results for feedstocks with no pretreatment other than autoclaving and for solubilization mediated by *C. thermocellum.* As observed in prior studies, *C. thermocellum* fermentation results in a dramatic decrease in viscosity. The magnitude of this decrease, however, is much larger starting with unpretreated feedstock than previously reported for pretreated feedstocks. LAOS measurements provide a detailed picture of the rheological fingerprint of the material. Viscosity measurements confirm the hypothesis that the physical character of corn stover slurries changes dramatically during fermentation by *C. thermocellum,* and indicate that the energy expended on overcoming slurry viscosity will be far less for partially fermented corn stover than for unfermented corn stover.

## Background

Cellulosic biomass is the primary product of photosynthesis and a desirable raw material to produce fuels and commodity chemicals [24]. Plant cell walls have, however, evolved to be difficult to deconstruct, and this recalcitrance is in large part responsible for the high cost of current conversion processes [12, 21]. To reduce this recalcitrance and, thus, increase the accessibility of the lignocellulose matrix to biological attack sufficiently to allow high yields of enzymatically mediated solubilization, pretreatment is widely thought to be necessary prior to biological processing using some combination of heat and chemicals [1, 16, 25]. The dominant paradigm for biological processing of cellulosic biomass, embodied in all six pioneer cellulosic ethanol plants, involves thermochemical pretreatment followed by the addition of fungal enzymes, and fermentation of soluble sugars by a non-cellulolytic microorganism [22, 39].

Thermophilic, anaerobic cellulolytic microbes such as *Clostridium thermocellum* (*Ruminoclostridium thermocellum*) are among the most effectively described biocatalysts at deconstructing cellulosic biomass [22, 28], and produce ethanol and other reduced metabolic end products as a result of non-respiratory metabolism. In light of these properties, *C. thermocellum* has received considerable attention for use in one-step consolidated bioprocessing (CBP) without added enzymes [20, 23, 26, 37]. This microbe has recently been found to be able to withstand milling during fermentation at intensities sufficient to allow very high solubilization of unpretreated senescent switchgrass [3]. Paye et al. [28] used the term “cotreatment” to refer to milling during fermentation. We herein refer to CBP in combination with cotreatment as C-CBP. Although at a nascent state of development and with many outstanding uncertainties, a scenario based on C-CBP with assumed research progress was shown to have potential to realize large cost savings compared to a current technology base case featuring thermochemical pretreatment and added enzymes [21]. The energy requirements for cotreatment—including but not limited to energy to overcome viscous dissipation—are not known and will be an important and perhaps decisive determinant of feasibility.

The rheological properties of lignocellulose slurries have received considerable attention with respect to thermochemical pretreatment followed by hydrolysis via fungal cellulase. Early work by Pimenova and Hanley [29, 30] using a helical impeller viscometer showed that pretreated corn stover (PCS) slurries exhibit shear-thinning behavior represented by a power law. Rheological properties were later shown to be reproducible across several different test methods and rheometer configurations and for a large range of solids concentrations [18, 36]. In general, viscosity increases with increasing solids concentration or particle size and decreases with increasing severity of pretreatment [38]. Studies have also looked at the rheology of PCS slurries as a function of saccharification using different reactor configurations, solids concentrations, particle sizes, and additives [4–6, 19, 32]. Slurry viscosities were seen to decrease the most during the first eight hours of reaction [4, 6], while yield stress decreased the most during the first day of reaction [32]. Both changes occurred regardless of the initial solids concentration. These data are consistent with the hypothesis that fermentation changes physical properties of lignocellulose slurries in ways that reduce milling energy. However, such property changes have not been reported for fermentation by *C. thermocellum.*

Rheometers with parallel plate or vane geometries are able to characterize small-particle slurries (typically sub millimeter), while a torque rheometer or specially designed apparatus is needed for larger particle slurries. For example, torque rheometry has been used to characterize the viscosity and yield stress of large-particle, high solid slurries [7, 33, 36]. Recently, Klingenberg et al. [17] designed a biomass rheometer that accommodates slurries at conditions relevant to commercial application including high solid concentrations and large particle sizes, as well as high temperature and low pH.

The motivating question for the present article is: *How does the rheological behavior of small*-*particle corn stover slurries (at various solids concentrations) change during the course of fermentation by C. thermocellum?* To address this question, we present experimental measurements of shear viscosity, as well as results of large amplitude oscillatory shear (LAOS) tests that provide a more detailed picture of the material’s rheological fingerprint. The present study is restricted to small particles due to the limitations of existing laboratory equipment and procedures. To our knowledge, this is the first study to characterize changes in the rheological properties of biomass slurries undergoing fermentation by *C. thermocellum.*

## Results

In this section, we report the results of three rheological experiments: (i) flow sweeps of corn stover slurries before and after fermentation; (ii) flow sweeps of samples taken over the course of fermentation; and (iii) large amplitude oscillatory shear (LAOS). During a *flow sweep*, the rheometer turns at steady state while torque is measured, and the torque and rotation rate data are used to infer strain rate, stress, and viscosity. In an *oscillatory shear* test, the rheometer oscillates at a specified frequency while measuring torque and rotation speed. All rheological experiments were conducted for corn stover slurries at various *insoluble solids concentrations* measured as described in the Methods section.

### Experiment 1: Rheology before and after fermentation

In this experiment, the rheological properties of unpretreated corn stover (with 0.2 mm maximum particle size) were examined without and with fermentation by *C. thermocellum*, a thermophilic, cellulolytic anaerobic bacterium. Steady shear flow sweeps were performed on slurries before and after fermentation. Slurries were prepared at four different insoluble solid concentrations $c$ = 10, 12.5, 15, and 17.5 wt.%. These concentrations span the capability range of the rheometer and are representative of conditions anticipated in an industrial process. Fermentation was carried out using *C. thermocellum*. After reaction, the solids remaining in the reactor were reconstituted to the same four concentrations as the unfermented slurries to allow for a direct comparison of viscosity before and after fermentation.

**Fig. 1:**
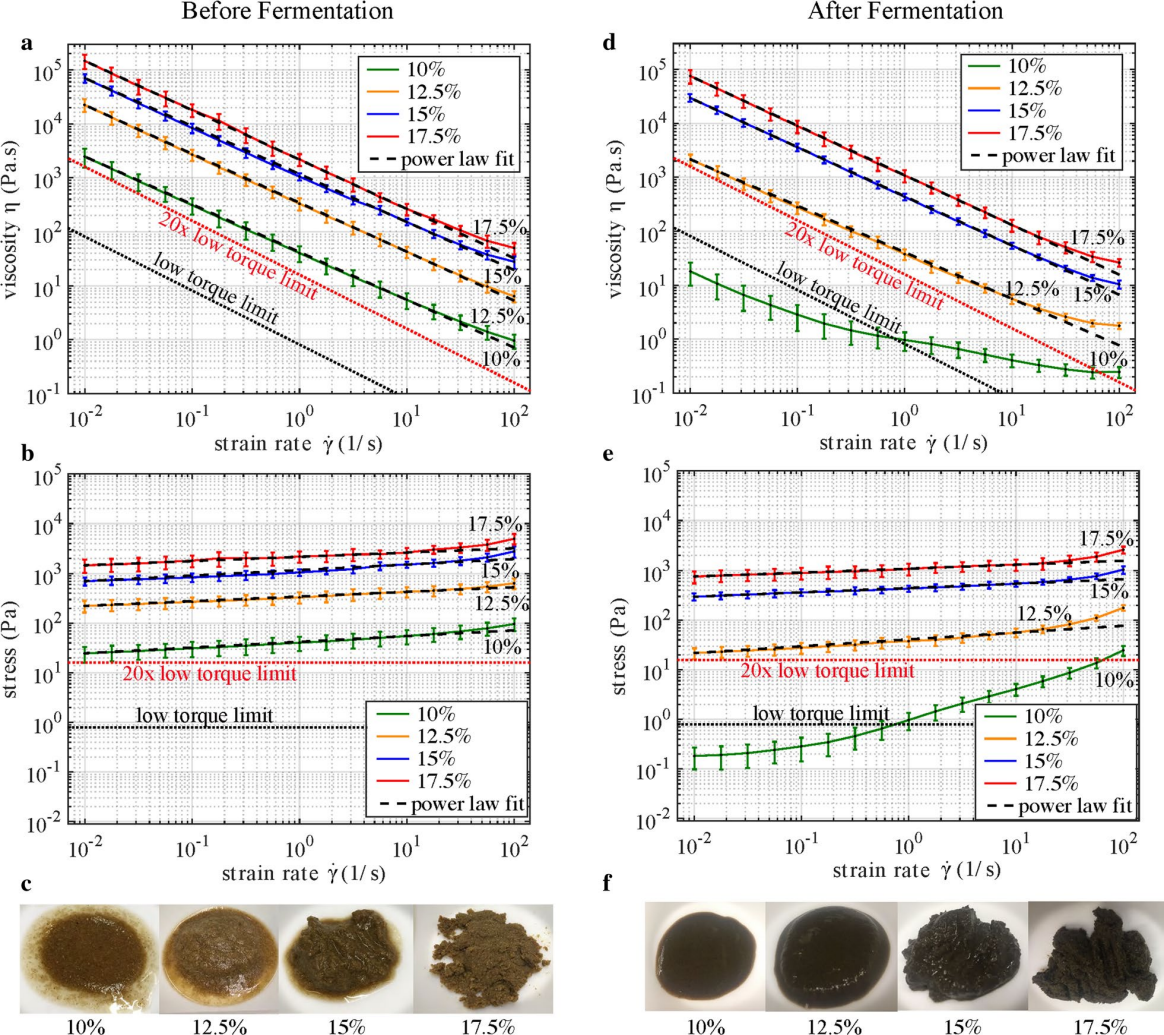
Unidirectional flow sweep results for corn stover slurries before fermentation (**a**–**c**) and after fermentation (**d**–**f**)

As illustrated in Fig. 1, the slurries ranged from being a pourable liquid to being a clay-like paste, depending on the solids concentration and state of fermentation. These extremes posed challenges to collection of repeatable shear sweep data: Low-solids-concentration slurries were liquid-like, so they had to be well mixed and then immediately loaded onto the rheometer to mitigate particle settling. High-solids-concentration slurries had no visible free water; so, care had to be taken to load the appropriate amount of material into the rheometer to maintain good contact between the top plate and the material yet prevent the top plate from squeezing water out of the slurry.

**Fig. 2:**
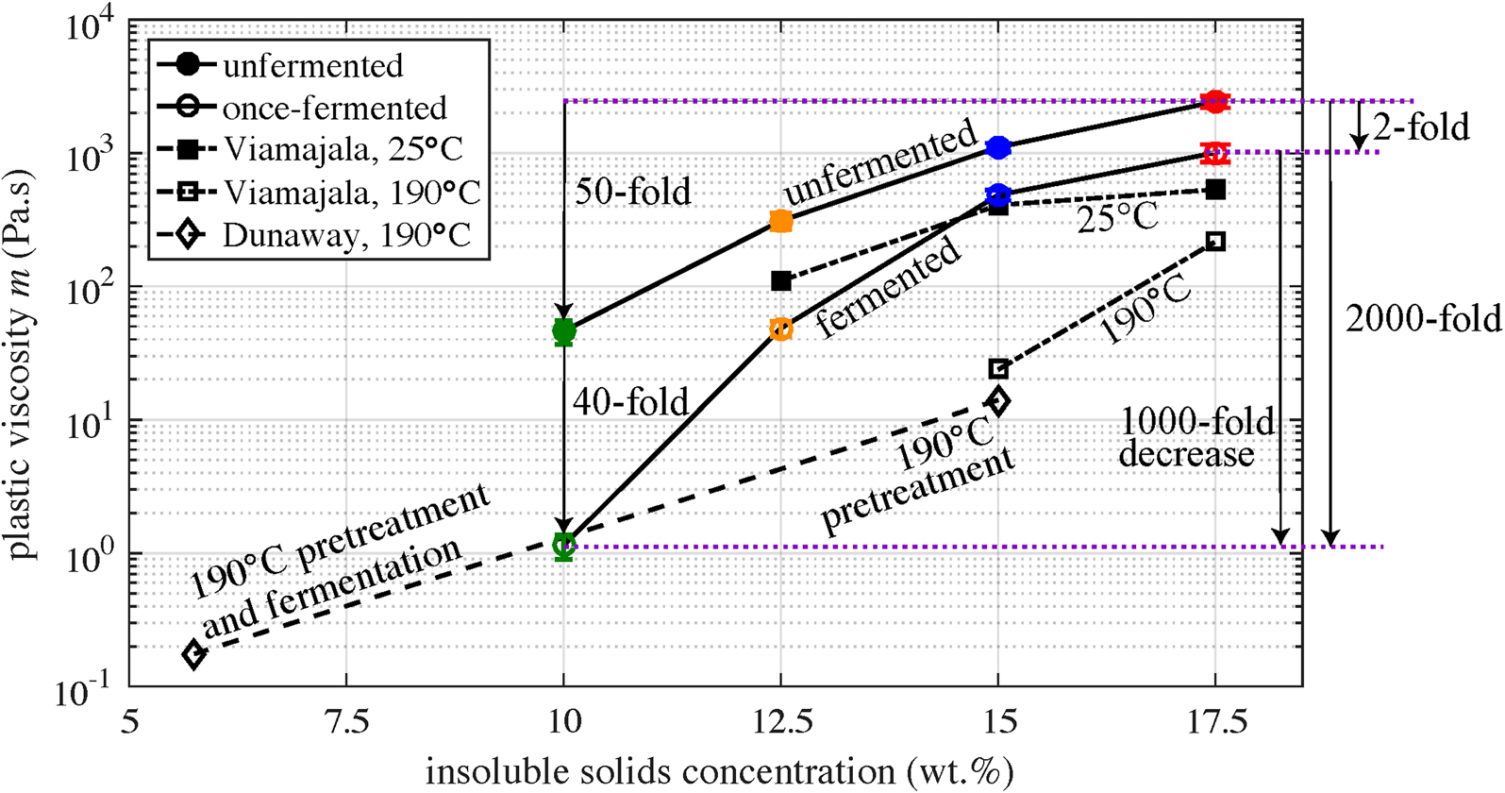
Plastic viscosity $m$ as a function of insoluble solids concentration $c$

Figure 1 reports viscosity and shear stress versus strain rate for flow sweep experiments, with power law fits displayed as dashed lines overlaid on the data. The black dotted lines represent the rheometer’s low-torque limit, 10 μNm, and the red dotted lines take into account manufacturing errors with 20 times this low-torque limit [9]. Viscosity is observed to decrease after fermentation and with lower solids concentration. For the range of strain rates considered, these corn stover slurries are well described by a power law constitutive model,

$$ \tau = m\dot{\gamma }^{n} $$

where $\tau$ is the shear stress (Pa), $\dot{\gamma }$ is the strain rate (1/s), $m$ is the plastic viscosity (Pa s), and $n$ is the power law index. Interestingly, all slurries have similar $n$ values of 0.10, indicative of highly shear-thinning behavior. Figure 2 reports the plastic viscosity of both the unfermented and fermented material. Also shown are literature data: Viamajala et al. [38] report data for corn stover slurries milled to − 80 mesh (0.177 mm) size and treated with dilute acid (1.5% H_2_SO4) at 25 °C and 190 °C. Dunaway et al. [6] provide data for before and after hydrolysis of a corn stover slurry with pretreatment via dilute acid (1.6% H_2_SO4) at 190 °C, hydrolysis for 170 h by fungal cellulase (*T. reesei*), and 0.03 mm particle size (Berson, personal communication).

It may be observed that the plastic viscosity of our fermented 10 wt.% corn stover slurry is 40-fold lower than that of its unfermented counterpart. At higher solids concentrations (for instance 17.5 wt.%), this viscosity reduction is less pronounced (twofold decrease). Plastic viscosity is observed to increase with increasing solids concentrations but plateau for very high concentrations. This “stacking” stems from frictional forces (between granular particles) dominating once the solids concentration is so high that the slurry contains no free water [38].

### Experiment 2: Rheology over the course of fermentation

In this experiment, flow sweeps and LAOS measurements were made on slurry samples drawn from the bioreactor during the course of fermentation to characterize the changes in rheology versus extent of fermentation. This section presents results of flow sweep tests, and the results of LAOS tests are presented in the next section. Duplicate fermentations for this experiment were carried out at an initial solids concentration of 8 wt.% (80 g/L), which is near the maximum allowable limit for batch cultures in light of initial mixing and sampling constraints in the laboratory bioreactor set-up employed. All slurry samples were concentrated twofold following fermentation and prior to rheological measurements to enable measuring viscosity above the low-torque limit of the rheometer. The solid concentrations referred to in this section are these twofold-concentrated values.

**Fig. 3:**
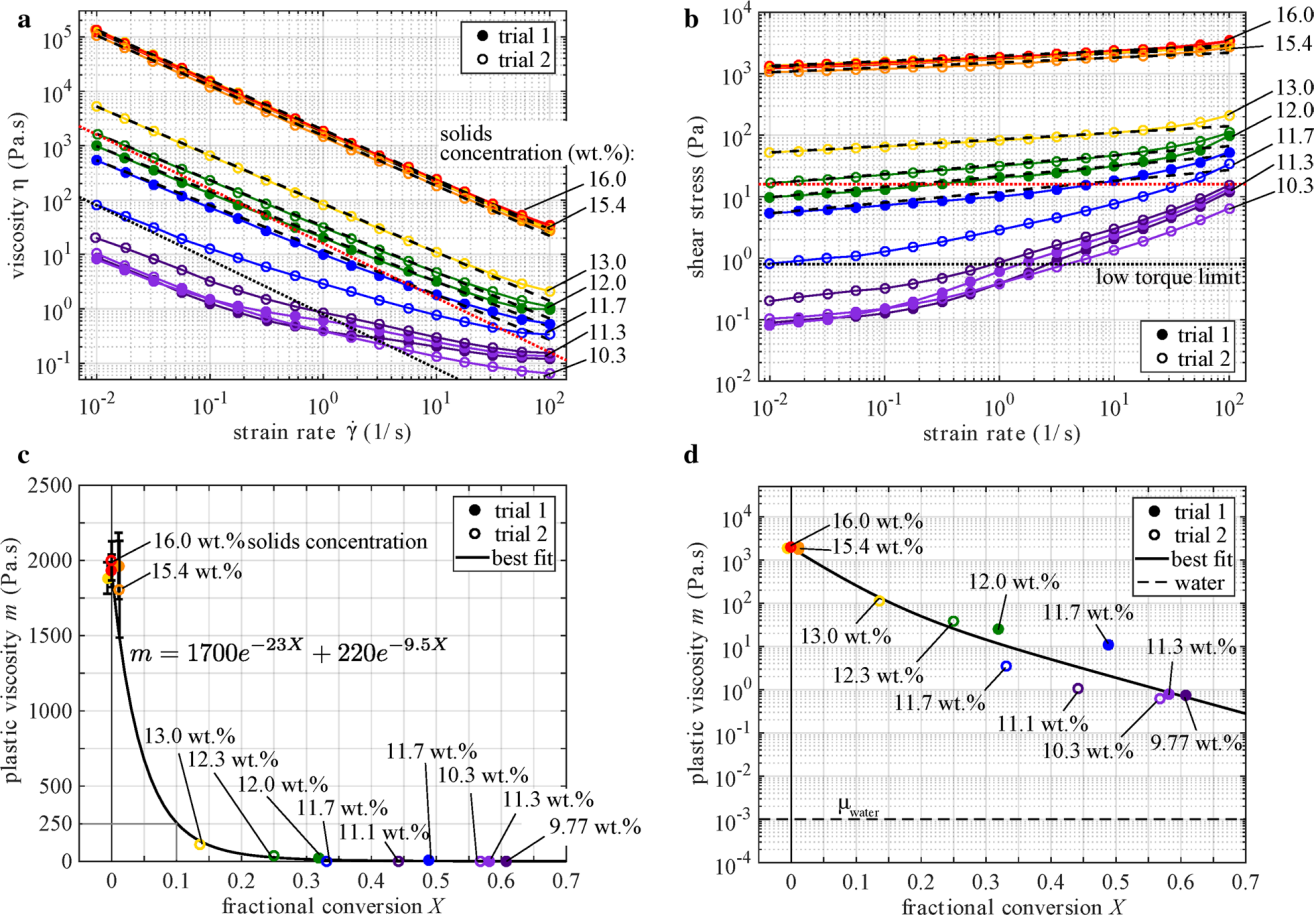
Viscosity (**a**) and stress (**b**) versus strain rate, and plastic viscosity (**c**, **d**) versus fractional conversion

Figure 3 presents the changes in viscosity as fermentation proceeds for a corn stover slurry with an initial insoluble solids concentration of 16 wt.%. Data for two separate fermentation trials are overlaid along with their power law fits. Plastic viscosity is plotted on a linear scale (c) and a log scale (d) as a function of fractional conversion $X$. Error bars represent 1 standard deviation. Data points for which error bars are not visible had standard deviations smaller than the symbols for the data. The solid concentrations at several fermentation time points are also indicated on Fig. 3a, b, with a lower solids concentration corresponding to a sample collected later in time. Consistent with the results presented in Fig. 1 (obtained at specified solids concentrations), results in Fig. 3a, b (at variable solids concentrations) exhibit several key features: shear-thinning behavior; viscosity well represented by a power law fit; and viscosity that decreases with decreasing solids concentration.

In Fig. 3c, d, plastic viscosity values are plotted versus fractional carbohydrate conversion on linear and log scales, respectively. The plastic viscosity falls from roughly 1920 to 1 Pa s over the course of the fermentation. Most of this nearly-2000-fold decrease takes place before 20% conversion, which corresponds to a fermentation time of approximately 2 days in these experiments. Interestingly, this 2000-fold decrease in viscosity is similar to that observed in Fig. 2 (therein considering a hypothetical fermentation run starting at 17.5 wt.% solids and reacting until a final concentration of 10 wt.% is reached). Here, the fermentation run started at 16 wt.% solids and ended at 10 wt.%; so, it is reasonable that the results of the two figures are in agreement.

The data were fit (via nonlinear least squares) with a double exponential curve, $m\, = \, 1700{\text{e}}^{{ - 23{\text{X}}}} \, + \,220{\text{e}}^{{ - 9.5{\text{X}}}}$, which illustrates the initial rapid decay of viscosity in the first stages of conversion $0 \le X \le 0.2$. The viscosity decreases eightfold (from 1920 to 250 Pa s) in the first 10% of conversion. A goodness-of-fit metric appropriate for a nonlinear regression such as this is the canonical angle $\psi$, which is the angle between the vector of data $\tilde{m}_{n}$ and the vector of model predictions, $m_{n}$. This angle can be computed via $\cos \psi \, = \,{{\left( { \mathop \sum \nolimits  m_{n} \tilde{m}_{n}  } \right)} \mathord{\left/ {\vphantom {{\left( { \mathop \sum \nolimits  m_{n} \tilde{m}_{n}  } \right)} {\left( {\sqrt {\mathop \sum \nolimits  m_{n}^{2} } \sqrt {\mathop \sum \nolimits  \tilde{m}_{n}^{2} } } \right)}}} \right. \kern-0pt} {\left( {\sqrt {\mathop \sum \nolimits  m_{n}^{2} } \sqrt {\mathop \sum \nolimits  \tilde{m}_{n}^{2} } } \right)}}$, where $\psi = 0^{ \circ }$ would indicate a perfect fit and $\psi = 90^{ \circ }$ would indicate that the model provides no explanation of the data. For these data and fit, the canonical angle is $\psi = 12^{ \circ }$. This is a reasonable fit; by analogy with linear data *y*(*x*) = * x * + * N*(0,σ^2^), the canonical angle depends linearly on the standard deviation σ (for $\psi < 20^{ \circ }$), and $\psi = 12^{ \circ }$ corresponds to *σ* = 0.122 and a *coefficient of determination* of *R*^2^ = 0.85.

### LAOS experiments

**Fig. 4:**
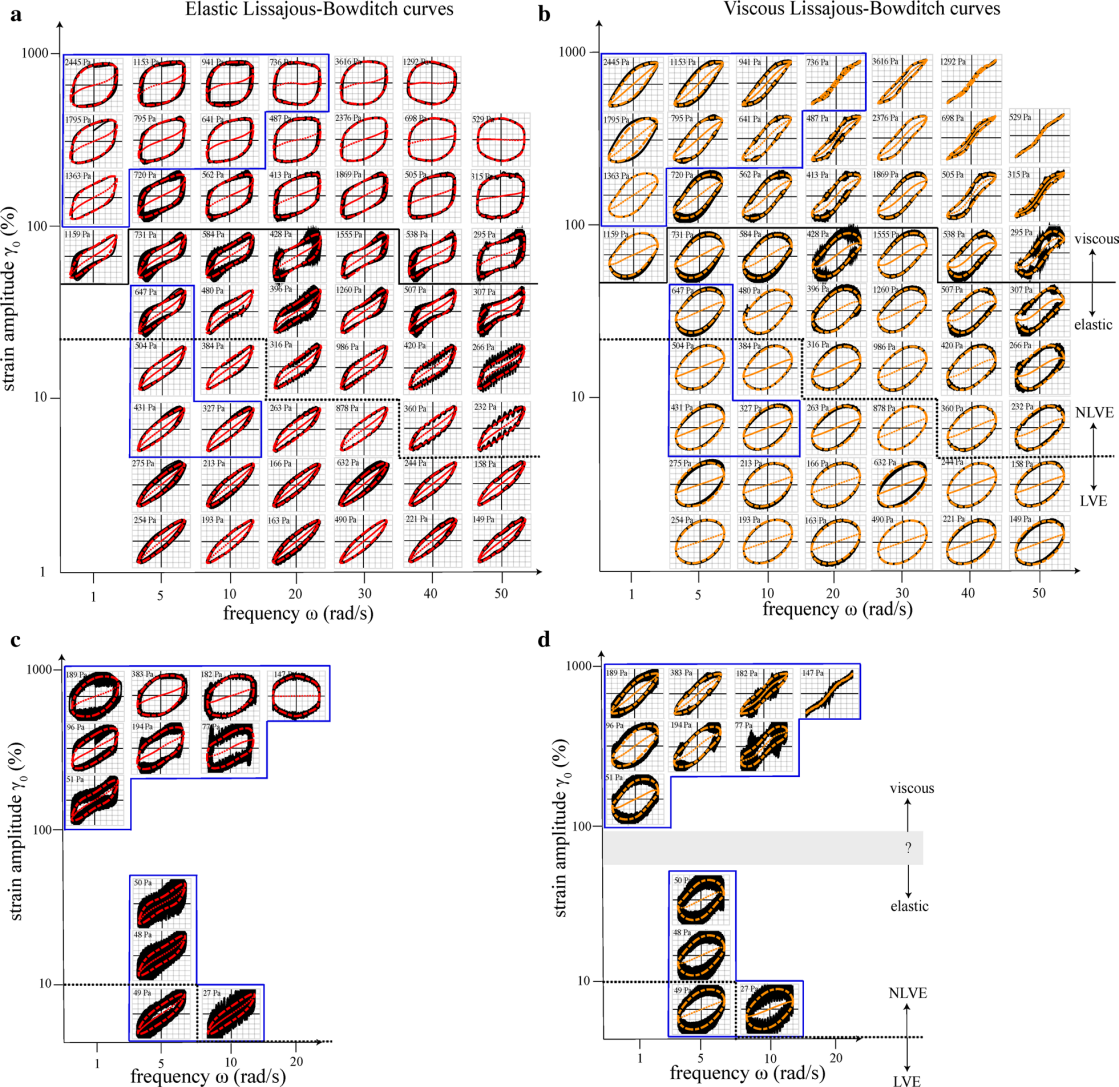
Elastic (**a**, **c**) and viscous Lissajous–Bowditch curves (**b**, **d**) for 16 and 13 wt.% slurries

Large amplitude oscillatory shear tests were conducted on the corn stover slurries sampled during *Experiment 2* to further probe their rheological properties. In each LAOS experiment, stress is measured while the material is harmonically strained, $\gamma \left( t \right) \, = \, \gamma_{0} { \sin }\left( {\omega t} \right)$. The stress response is plotted as stress versus strain (elastic Lissajous–Bowditch (ELB) curve) or stress versus strain rate (viscous Lissajous–Bowditch (VLB) curve). A perfectly elastic body would have a straight-line ELB curve and a circular VLB curve, and vice versa for a perfectly viscous fluid. Each subfigure in Fig. 4 contains a family of L–B (Lissajous–Bowditch) curves, with each curve centered around its frequency $\omega$ (rad/s) and amplitude $\gamma_{0}$ (%) in a Pipkin map [31]. LAOS data are typically filtered using a three-odd-term Fourier series [8]:

$$ \sigma \left( t \right)\, = \,\sigma^{\prime}\left( t \right)\, + \,\sigma^{\prime\prime}\left( t \right)\, = \,\mathop \sum \limits_{n = 1,3,5} \gamma_{0} G^{\prime}_{n} \sin n\omega t\, + \, \gamma_{0} G^{\prime\prime}_{n} \cos n\omega t$$

where $\sigma^{\prime}\left( t \right)$ and $\sigma^{\prime\prime}\left( t \right)$ represent the elastic and viscous stress contributions, and the $G_{n}^{'} \left( {\gamma_{0} ,\omega } \right)$ and $G_{n}^{''} \left( {\gamma_{0} ,\omega } \right)$ are the viscoelastic moduli.

Figure 4 shows ELB and VLB curves for corn stover slurries at time = 0 h (16 wt.% solids, $$ X = 0 $$, *m* = 1920 Pa) (a, b) and time = 44 h (13 wt.% solids, $$ X = 0.14 $$, *m* = 112 Pa) (c, d) after the start of fermentation. Each curve’s raw data and filtered data are plotted in black and in color, respectively. The maximum stress $$ \tau_{ \hbox{max} } $$ (Pa) is shown above each curve. The elastic stress $$ \sigma^{\prime}\left( t \right) $$ or viscous stress $$ \sigma^{\prime\prime}\left( t \right) $$ is plotted in dotted lines. In (a) and (b), the dotted line partitions the linear viscoelastic region and the nonlinear viscoelastic region. Also, the solid black line indicates the crossover line that delineates the predominantly elastic region (below) and the predominantly viscous region (above). Figure 4a, b show ELB and VLB curves, respectively, for a 16 wt.% solids “hour 0” slurry, which is essentially unfermented. These Pipkin maps show three distinct regions: linear viscoelastic (LVE); nonlinear viscoelastic (NLVE) yet still elastically dominated; and viscous dominated. The LVE region (defined herein as that for which the third- and fifth-order Fourier coefficients are less than 5% of the first-order coefficients) occurs for small strain amplitudes, with the cutoff amplitude depending on the frequency (as shown in Fig. 4) but being roughly $$ \gamma_{0} \,\underset{\raise0.3em\hbox{$\smash{\scriptscriptstyle\thicksim}$}}{ < }  \, 10\% $$. In the LVE region, the narrowly elliptical shape of the ELB curves indicates that elastic behavior dominates at these low strain amplitudes.

In the NLVE region, the shapes of the L–B curves illustrate the complex rheology of the slurry. For example, the inward curvature of the viscous stress (dashed lines in the VLB curves) suggests that the deviations from linearity are due to shear-thinning, which reaffirms the results in *Experiment 1*. Also, in the NLVE region, both the ELB and VLB curves are tilted near the edges, as previously observed by [35] for pretreated, unfermented slurries at similar solids concentrations. For moderate strain amplitudes $10\% \,{ \lesssim }\,\gamma_{0} \,{ \lesssim }\,100{\text{\% }}$, the NLVE region is characterized by predominantly elastic behavior.

**Fig. 5:**
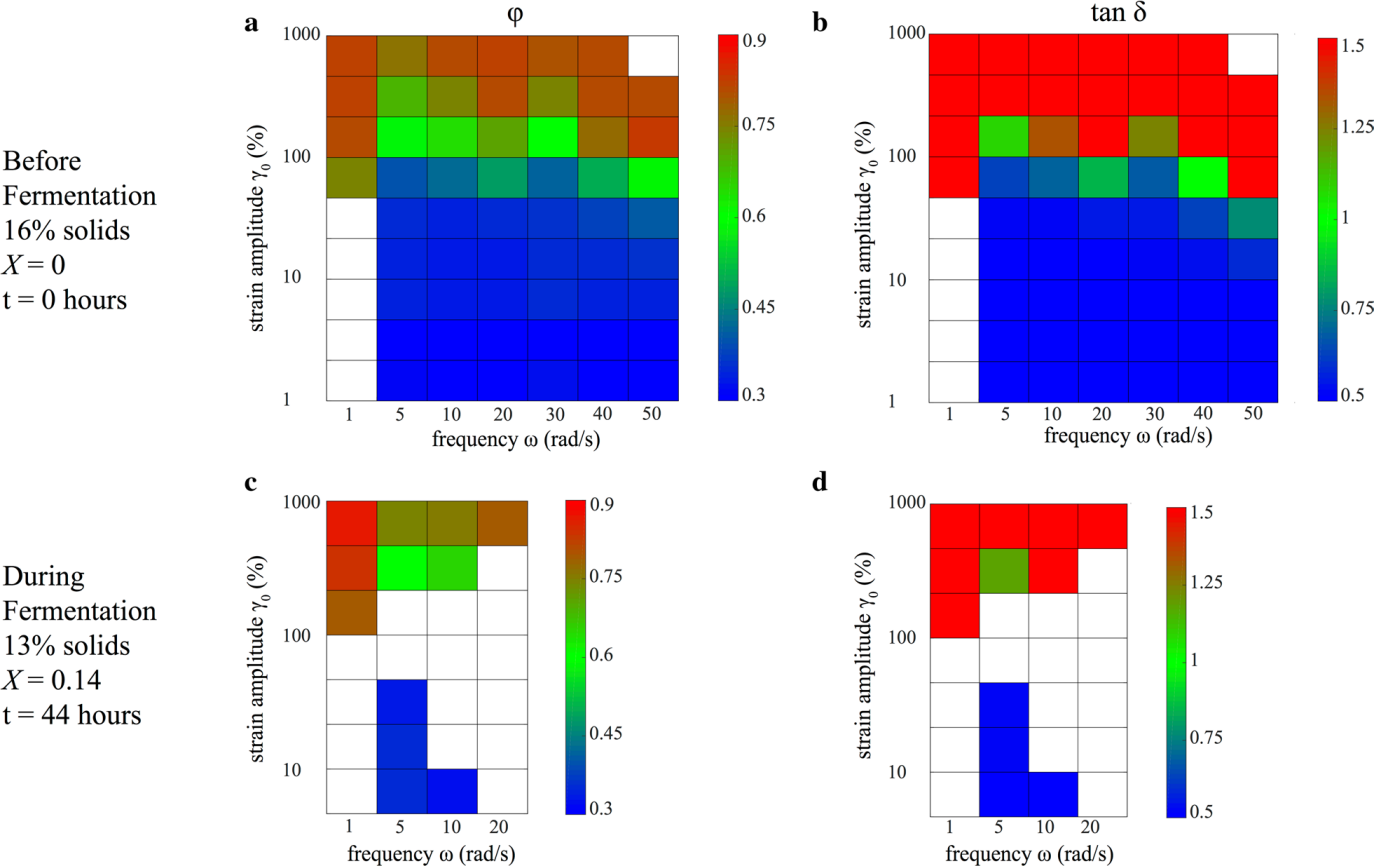
Color maps of the perfect plastic dissipation ratio $\phi$ (**a**, **c**) and phase angle $\tan \delta = G_{1}^{''} /G_{1}^{'}$ (**b**, **d**)

The transition from elastic to viscous behavior is given where the phase amplitude $\tan \delta = G_{1}^{''} /G_{1}^{'}$ is unity. Figure 5b shows $\tan \delta = 1$ at roughly $\gamma_{0} \approx 100\%$. It is interesting to note that the noisiest data occur on this elastic–viscous crossover boundary; this is not surprising given that for smaller strain amplitudes, the rotor is moving too little to disrupt the matrix of solids particles, whereas for much larger strain amplitudes, the matrix is disrupted so much that the displacements of individual particles are averaged out.

For high strain amplitudes and frequencies, the flow is dominated by viscous effects. In this region, some VLB curves are observed to self-intersect, which is indicative of a strong elastic nonlinearity caused by a viscoelastic stress overshoot, essentially meaning that the rate at which the slurry is unloading stress is faster than its rate of deformation [10]. Such nonlinearities are typically present due to reversible changes in the material’s disposition (with one possibility being that the slurry’s entrained water is being squeezed out upon deformation). At very high amplitudes and frequencies, the square-shaped elastic curves show that the slurry is plastically deforming.

Figure 4c, d shows the corresponding LAOS data for a 13 wt.% solids “hour 44” slurry. This 13 wt.% solids sample had visible free water, making data collection quite challenging. Most of the raw data were too noisy to be included in the Pipkin map, but with the available data, a few comparisons can be made. The LVE–NLVE transition occurs at a lower strain amplitude for the “hour 44” slurry, and this transition is due to its shear-thinning behavior, similar to the “hour 0” slurry. Overall, the shapes of the L–B curves are surprisingly similar for the “hour 0” and “hour 44” slurries, given that the steady shear viscosity has fallen by a factor of seventeen.

Having characterized the nonlinear response of slurries using Lissajous–Bowditch curves, parameters like the *perfect plastic dissipation ratio* $\phi$ and *phase angle* $\delta$ were computed to assess yielding behavior. Figure 5 displays a checkerboard plot of $\phi$ and $\tan \delta$, with each square in Fig. 5 corresponding to a pair of Lissajous–Bowditch curves in Fig. 4. Like Fig. 4, Fig. 5 displays data for corn stover slurries at time = 0 h (16 wt.% solids, $X = 0$, *m* = 1920 Pa) and time = 44 h (13 wt.% solids, $X = 0.14$, *m* = 112 Pa) (c, d) after the start of fermentation, plotted on a Pipkin space. White squares on the Pipkin space indicate that the data were too noisy to be reported. The perfect plastic dissipation ratio compares the actual energy dissipated by an L–B curve to its theoretical value [11],

$$ \phi \, = \, \frac{{\pi \gamma_{0}^{2} G^{\prime\prime}_{1} }}{{4\gamma_{0} \sigma_{\text {max}} }}\, = \, \frac{{\pi \gamma_{0} G^{\prime\prime}_{1} }}{{4\sigma_{\text {max}} }} $$

where $\gamma_{0}$ is the strain amplitude (unitless), $G_{1 }^{''}$ is the loss modulus (Pa), and $\sigma_{ \text{max} }$ is the maximum stress (Pa). Graphically, $\phi$ is the ratio of the area inside the L–B curve to the area of the smallest rectangle that can be drawn around the L–B curve, with $\phi = 0$ indicating a perfectly elastic response, $\phi \, = \,\frac{\pi }{4} \,\sim \,0.785$ corresponding to a Newtonian fluid, and $\phi = 1$ indicating a perfectly plastic response.

In Fig. 5a, the 16 wt.% “hour 0” slurry shows $0.30\, \le \,\phi \, \le \,0.83$, with this upper limit of $\phi = 0.83$ being between Newtonian and perfectly plastic. The slurry transitions from exhibiting elastoplastic behavior ($\phi \le \frac{\pi }{4} )$ to exhibiting pseudoplasticity $\left( {\phi \ge \frac{\pi }{4} } \right)$ at roughly $\gamma_{0} \approx 100$%, which is consistent with the elastic–viscous crossover at $\tan \delta = 1$. In Fig. 5c, the 13 wt.% “hour 44” slurry’s perfect plastic dissipation ratios range from $0.32 \le \phi \le 0.87$, which again is remarkably similar to the “hour 0” slurry. Like its unfermented counterpart, it did not exhibit perfect plastic yielding, but showed elastoviscoplastic deformation.

## Discussion

Prior literature involving thermochemical feedstocks and fungal cellulase has reported a ~ 80-fold decrease in viscosity upon enzymatic hydrolysis mediated by fungal cellulase [6] (Fig. 2), whereas herein we report a 2000-fold decrease upon fermentation by *Clostridium thermocellum* (Fig. 3). It is important to note that [6] characterized viscosity changes during hydrolysis of corn stover slurries subjected to thermochemical pretreatment, whereas we used unpretreated corn stover. As a result, the initial viscosity was much lower in the study of [6] than in our study. An estimate of the viscosity of Dunaway’s unpretreated material can be made by considering the data from [38] (Fig. 2), which show a ~ 18-fold decrease in viscosity due to dilute-acid pretreatment. Multiplying 18- and 80-fold changes, a 1440-fold decrease in viscosity due to both pretreatment and hydrolysis may be calculated. This is similar to the 2000-fold decrease observed herein, albeit with a different biocatalyst.

It is of interest to compare the impact of particle size and biological conversion on the change in viscosity observed during fermentation. This comparison is not straightforward, however, because these factors are nonlinearly coupled. Comparing the 17.5 wt.% fermented, 10 wt.% unfermented, and 10 wt.% fermented data points in Fig. 2, one observes a 50-fold reduction in viscosity due to solids concentration and a 40-fold decrease due to fermentation. However, comparing the 17.5 wt.% unfermented, 17.5 wt.% fermented, and 10 wt.% fermented data points, one observes a twofold reduction in viscosity due to fermentation and a 1000-fold decrease due to solids concentration. So, in either case, the solids concentration has a larger effect than fermentation does, but how much larger depends on the basis of comparison.

A cautious comparison can be made with respect to the effect of fermentation versus the effect of dilute-acid pretreatment using the data in Fig. 2. Comparing Viamajala 25 °C baseline and 190 °C pretreatment data at 17.5 wt.% solids, one observes a 2.5-fold reduction in viscosity due to pretreatment; this is similar to our observed reduction in viscosity due to fermentation. However, at 15 wt.% solids, 190 °C pretreatment causes a much larger reduction in viscosity compared to the reduction due to fermentation. One hypothesis for this difference is that, whereas dilute-acid pretreatment preferentially removes hemicellulose, biological attack (by either commercial cellulase or thermophilic bacteria) solubilizes cellulose and hemicellulose to a similar extent [28].

It may be observed from Fig. 3c that the viscosity at $X = 0.3$ is negligible compared to its initial value. Thus, if mechanical treatment could be withheld until $X = 0.3$, about half the maximum achieved in our experiments, the energy required to overcome viscous energy dissipation during milling would be vastly reduced. With respect to mixing fermentation broth, as distinct from milling, both turbulent and laminar flow could be present in fermentors containing reacted solids depending on the impeller speed and extent of reaction. Consider an industrial-scale reactor with a characteristic length scale $l$ of 1 m and a characteristic flow speed $v$ of 1 m/s (e.g., flow created by an impeller) and for ease of calculation, assume that the slurry has a density $\rho$ similar to water (1000 kg/m^3^). Given that measured viscosities range from *μ* = 10^5^ to 10^−1^ Pa s, the Reynolds number (${\text{Re}}\, = \,{{\rho vl} \mathord{\left/ {\vphantom {{\rho vl} \mu }} \right. \kern-0pt} \mu }$) of such a flow would range from 10^−2^ up to 10^4^, indicating laminar through turbulent flows. Future work will need to characterize the energy dissipation due to mixing in a representative reactor through both the laminar and turbulent regimes.

Making rheological measurements with biomass poses a number of challenges [36]. The low- and high-torque limits of the rheometer we used precluded measurements with very-low-solids (< 10 wt.%) or very-high-solids (> 20 wt.%) slurries. Shear rates were limited on the low end to 10^−2^ s^−1^ due to a combination of the low speed limit of the rheometer and stick-slip behavior of the slurries at low speeds, which resulted in very large error bars at these speeds. Unfortunately, this low speed limit (10^−3^ rad/s) prevented yield stress measurements from being carried out. On the high end, shear rates above 10^2^ s^−1^ resulted in $\tau \sim \dot{\gamma }^{2}$, indicating secondary flows and turbulence [9]. Despite these challenges, the collected data were repeatable and span a wide range of viscosities and shear rates.

This work provides a foundation for studying the rheology of more industrially realistic biomass slurries with large-particle and high-solid loadings undergoing fermentation with *C. thermocellum*. Future work is planned to assess the overall energy requirements for milling partially fermented solids as well as the relative importance of viscous energy dissipation and particle breakage determinants of energy requirements. Large-scale biomass slurries could be analyzed, say, using the methods outlined in [17, 27]. Other biomass feedstocks could also be considered. These viscosity and power measurements will inform future techno-economic modeling as well as the design of industrial biomass conversion plants that employ cotreatment.

## Conclusions

The rheology of corn stover during fermentation by *Clostridium thermocellum* was considered. Steady shear viscosity fit well to a power law model and exhibited shear-thinning. During the course of fermentation, plastic viscosity decreased by a factor of 2000, with the first eightfold decrease in the first 10% of carbohydrate conversion. This dramatic decrease suggests radical changes in the slurry’s physical properties, which has positive but not definitive implications for the feasibility of cotreatment. LAOS experiments revealed similar elastoviscoplastic rheological fingerprints for both unfermented and partially fermented slurries, with plastic deformation at large strain amplitudes and deviations from linearity due to shear-thinning.

## Methods

### Feedstock preparation

#### Feedstock milling

Corn stover, ground to pass through a 5-mm screen, was received as a gift from the US Department of Energy Great Lakes Bioenergy Research Center (GLBRC corn stover UW 2009). This material was subsequently milled with a Retsch ZM 200 centrifugal mill (Retsch, Haan, Germany) using a 0.2-mm aperture ring sieve. This 0.2-mm particle size was chosen to enable reliable parallel plate rheological measurements and to allow for comparison to literature data from [38]. The milled corn stover was stored in sealed containers in the dark until use. This feedstock was used as the starting material for all experiments.

#### Slurry solids concentrations

Corn stover slurries for rheological measurements were prepared with several insoluble solids concentrations, $c$, defined as the ratio between the dry mass of the biomass $\left( {1 - p} \right)m_{\text{b}}$ to the total mass of biomass and water,

$$ c = \frac{{\left( {1 - p} \right)m_{\text{b}} }}{{m_{\text{b}} + m_{\text{a}} }}$$

where $p$ is the percent moisture content of the biomass, $m_{\text{b}}$ is the mass of the biomass, and $m_{\text{a}}$ is the mass of added water. The factor $\left( {1 - p} \right)$ accounts for the moisture content of the feedstock, which is $pm_{\text{b}}$. The value of $p$ was measured using an MX-50 moisture analyzer (A&D company, San Jose, CA), with measurements made in triplicate just prior to preparing the slurries.

### Fermentation

#### Organism and cultivation medium

The strain used for all bioreactor experiments was *C. thermocellum* strain LL1043 (M1570). This is a pH-auxostat-adapted strain with deletions for *hpt*, *ldh* and *pta*, as described by [2].

The cultivation medium used here is a variation of Medium for Thermophilic Clostridia (MTC) as described in [15] with 2 g/L urea as nitrogen source, 4× increased vitamins (final concentrations; 80 mg/L pyridoxamine dihydrochloride, 16.0 mg/L para-aminobenzoic acid (PABA), 8 mg/L d-biotin and 8 mg/L vitamin B_12_), and 5× increased trace elements (final concentration; 5.0 mg/L MnCl_2_·4H_2_O, 2.5 mg/L ZnCl_2_·6H_2_O, 0.5 mg/L CoCl_2_·6H_2_O, 0.5 mg/L NiCl_2_·6H_2_O, 0.5 mg/L CuSO_4_·5H_2_O, 0.5 mg/L H_3_BO_3_ and 0.5 mg/L Na_2_MoO_4_). The corn stover, together with the bulk of the water, was autoclaved in the bioreactor. The various medium components were added to the bioreactor containing the corn stover slurry by filter sterilization as separate solutions, as described in [13].

#### Bioreactor cultivation and inoculum preparation

All bioreactor experiments described here were done in a 2.2-L Sartorius Aplus bioreactor cultivation system at a working volume of 2 L, with pH controlled at 7.0 using a gel-filled pH probe (Mettler-Toledo, Billerica MA) combined with the automatic addition of 4 M KOH. The corn stover slurry was initially stirred at 500 rpm prior to and after inoculation; once growth initiated (as evident from gas formation) stirring was decreased to 300 rpm. The incubation temperature was maintained at 55 °C, and the off-gas condenser was at kept at 4 °C. Gas production was monitored with a milligas flow meter (Ritter, Hawthorne NY) filled with 0.5 M HCl. Total volumetric gas production data was recorded by accompanying Rigamo software.

The inoculum was prepared by culturing *C. thermocellum* LL1043 on 50 g/L cellulose (Avicel PH105) in a pH-controlled bioreactor, and harvesting at peak volumetric gas production rate by storing 50 mL cell culture aliquots in 125 mL serum bottles at − 80 °C. All fermentations reported here were inoculated with one of these 50-mL cell culture aliquots.

#### Determining carbohydrate solubilization

Solubilization of the corn stover was quantified by the fractional conversion:

$$ X\left( t \right) = 1 - {\raise0.7ex\hbox{${m_{\text{c}} \left( t \right)}$} \!\mathord{\left/ {\vphantom {{m_{\text{c}} \left( t \right)} {m_{\text{c}} \left( {t = 0} \right)}}}\right.\kern-0pt} \!\lower0.7ex\hbox{${m_{\text{c}} \left( {t = 0} \right)}$}} $$

where $t$ is the time and $m_{\text{c}}$ is the total mass of carbohydrates (g), which is the sum of the masses of glucan, xylan, and arabinan present in the sample. These constituents were determined via *quantitative saccharification* (QS), performed in triplicate using the NREL protocol [34, 14]. Herein, we refer to solubilization and fractional conversion interchangeably.

### Rheometry

Rheological measurements were carried out using a TA Instruments AR 2000ex rheometer with 40-mm diameter, cross-hatched parallel plates (TA Instruments, New Castle, DE). A 48-mm inner diameter, 6-mm high retaining collar, similar to that in [36], was fabricated and attached to the bottom plate to prevent material ejection. Strain-controlled shear data were collected by carrying out flow sweeps from $\dot{\gamma }$ = 100 to 0.01 s^−1^ using three points per decade, a gap height of 1.5 mm, and a sampling time of 15 s per data point. Each trial was replicated at least five times with a freshly loaded sample.

Strain-controlled LAOS measurements were performed with a test matrix of frequencies 1 ≤ $\omega$ ≤ 50 rad/s and strain amplitudes 0.01 ≤ $\gamma_{0 }$ ≤ 10 (unitless), with four points per decade. These ranges were chosen based on initial trials, because raw stress signals outside this range were unacceptably noisy. Raw data were post-processed with MITlaos [8] using a maximum Chebyshev harmonic of 5 to yield filtered LAOS data. Data were excluded from the study if the stress/strain signals were too noisy (typically at low frequencies and amplitudes) to enable a reliable Chebyshev fit.

### Experiment 1: Rheology before and after fermentation

The milled feedstock was used to prepare corn stover slurries at 10, 12.5, 15, 17.5 wt.% insoluble solids concentrations. The viscosity of these unfermented slurries was measured via flow sweeps (as described in §4.3). The milled feedstock was also used to prepare a slurry at 10 wt.% (100 g/L) initial solids concentration for subsequent fermentation (as described in §4.3). This slurry was fermented for 188 h and harvested after gas production ceased; the final solids concentration was 5.9 wt.% (59 g/L). The whole reactor content was harvested by spinning down the broth at 10,000×*g* at 4 °C. A representative sample of this material was analyzed for residual carbohydrates and dry weight by further spinning down and drying the pellet at 60 °C, followed by determination of glucan, xylan, and arabinan content by QS. Another representative sample was spun down (to roughly 19 wt.% solids) and then re-suspended with water to make once-fermented slurries (at 10, 12.5, 15, 17.5 wt.% solids), which were then used for viscosity measurements.

### Experiment 2: Rheology during the course of fermentation

The following experiment was performed in duplicate: The milled feedstock was used to prepare a corn stover slurry at 8 wt.% (80 g/L) initial solids concentration for subsequent fermentation. The fermenting slurry was sampled at 6–7 time points, including time *t* = 0. For each time point, a 130-mL sample was transferred to a beaker on a stir plate. Triplicate aliquots of 10 mL were taken for QS; the remainder was used immediately for rheological measurements and for determining the solids concentration. These aliquots were spun down at 7800×*g*, rinsed with an equal volume DI water once and spun down again, after which the residues were recovered and dried at 60 °C for at least 5 days, followed by determination of glucan, xylan, and arabinan content by QS. Rheological measurements included flow sweeps and LAOS.

Prior to rheological measurements, the harvested slurry samples were concentrated 2× by removing half the volume of water (by spinning down at 10,000×*g* at 4 °C). For example, an 8 wt.% sample was concentrated to 16 wt.%. This adjustment was necessary, because the fermentation required concentrations less than 10 wt.% but the rheological measurements required concentrations greater than 10 wt.%. Solid concentrations greater than 10 wt.% are prohibitive for fermentation due to difficulties in mixing and sampling at early time points. Solid concentrations less than 10 wt.% (for this particle size) would result in viscosity measurements below the rheometer’s low-torque limit. Hence, the effective solution for obtaining reliable rheological results during the course of fermentation was to remove half the water from the slurry samples.

## Abbreviations

CBP: consolidated bioprocessing
C-CBP: consolidated bioprocessing in combination with cotreatment
ELB: elastic Lissajous-Bowditch
LAOS: large amplitude oscillatory shear
LVE: linear viscoelastic
NLVE: nonlinear viscoelastic
PCS: pretreated corn stover
VLB: viscous Lissajous-Bowditch
